# New Appliances and Things Medical

**Published:** 1905-05-20

**Authors:** 


### NEW APPLIANCES AND THINGS MEDICAL.

[We shall be glad to receive, at our Office, 28 & 29 Southampton Street, Strand, London, W.C., from the manufacturers, specimens of all new preparations and appliances which may be brought out from time to time.]

### DRIED MILK (COW AND GATES BRAND).

(West Surrey Central Dairy Company, Guildford.)

The preparations before us are powders produced by the Just Hatmaker process. The powders are essentially of three kinds, according to the fat they contain, and are called "separated" milk powder, "half cream" milk powder, and "full cream" milk powder respectively. They are made up in different coloured cardboard cases; the cases containing the separated and half-cream milk powders having printed on their side an analysis of the respective powder. Packets were supplied of two sizes, corresponding in the cases of the full and half-cream milk powder to the solid matter obtained from three pints in the one case and three quarts in the other, and in the separated milk powder to four pints and four quarts. The directions given are that dilution should be effected with hot, but not boiling, water, and the quantity of water to be given to a given weight of powder is based upon the fact that the whole packet corresponds to the solid matter of a known (given) volume of milk. The milk when made according to directions is a liquid very similar to ordinary milk, and has a pleasant flavour. If prepared according to the directions it will correspond to a separated, half, or whole cream milk according to the powder used. The milk thus prepared adapts itself well to cooking purposes. We think these milk powders should have a large sphere of usefulness.

### HALL'S WINE (TONIC).

(Stephen Smith and Co., Bow, London.)

We have received a sample bottle of this well-known tonic preparation. It consists essentially of a wine with which are incorporated the active principles of the leaves of the erythroxylon coca. As is well known to pharmacologists these leaves contain not only the alkaloid cocaine, but also other alkaloids in smaller quantities yielding like cocaine ecgonin upon chemical decomposition. The action of these substances is imperfectly known, but extracts made from the leaves, and the leaves themselves, are more potent as nervine tonics than the quantity of cocaine which they contain when given alone. The stimulating action of cocaine upon the central nervous system is well known, but it is not perhaps so well recognised that its stimulating action is a descending one affecting primarily the cerebrum, and to a much less degree the medulla and spinal cord. We think Hall's wine is an agreeable and efficacious way of prescribing this remedy, but it should be remembered that cocaine is one of those drugs production of "habit," and its use in any form requires careful supervision at the hands of the medical man. The alcoholic strength of the preparation before us is approximately equal to that of an average port.

